# The association between comorbidities and pain, physical function and quality of life following hip and knee arthroplasty

**DOI:** 10.1007/s00296-015-3211-7

**Published:** 2015-01-14

**Authors:** W. F. Peter, J. Dekker, C. Tilbury, R. L. Tordoir, S. H. M. Verdegaal, R. Onstenk, M. R. Bénard, S. B. Vehmeijer, M. Fiocco, H. M. Vermeulen, H. M. J. van der Linden-van der Zwaag, R. G. H. H. Nelissen, T. P. M. Vliet Vlieland

**Affiliations:** 1 Department of Orthopaedics J11, Leiden University Medical Center, P.O. Box 9600, 2300 RC Leiden, The Netherlands; 2 Amsterdam Rehabilitation Research Center | Reade, Amsterdam, The Netherlands; 3 Department of Psychiatry, Department of Rehabilitation Medicine, EMGO Institute, VU University Medical Center, Amsterdam, The Netherlands; 4 Department of Orthopedics, Rijnland Hospital, Leiderdorp, The Netherlands; 5 Department of Orthopedics, Groene Hart Hospital, Gouda, The Netherlands; 6 Department of Orthopedics, Reinier de Graaf Hospital, Delft, The Netherlands; 7 Department of Medical Statistics and Bioinformatics, Leiden University Medical Center, Leiden, The Netherlands

**Keywords:** Osteoarthritis, Comorbidity, Surgery

## Abstract

The aim of the study was to examine the relationship between comorbidities and pain, physical function and health-related quality of life (HRQoL) after total hip arthroplasty (THA) and total knee arthroplasty (TKA). A cross-sectional retrospective survey was conducted including 19 specific comorbidities, administered in patients who underwent THA or TKA in the previous 7–22 months in one of 4 hospitals. Outcome measures included pain, physical functioning, and HRQoL. Of the 521 patients (281 THA and 240 TKA) included, 449 (86 %) had ≥1 comorbidities. The most frequently reported comorbidities (>15 %) were severe back pain; neck/shoulder pain; elbow, wrist or hand pain; hypertension; incontinence of urine; hearing impairment; vision impairment; and cancer. Only the prevalence of cancer was significantly different between THA (*n* = 38; 14 %) and TKA (*n* = 52; 22 %) (*p* = 0.01). The associations between a higher number of comorbidities and worse outcomes were stronger in THA than in TKA. In multivariate analyses including all comorbidities with a prevalence of >5 %, in THA dizziness in combination with falling and severe back pain, and in TKA dizziness in combination with falling, vision impairments, and elbow, wrist or hand pain was associated with worse outcomes in most of the analyses. A broad range of specific comorbidities needs to be taken into account with the interpretation of patients’ health status after THA and TKA. More research including the ascertainment of comorbidities preoperatively is needed, but it is conceivable that in particular, the presence of dizziness with falling, pain in other joints, and vision impairments should be assessed and treated in order to decrease the chance of an unfavorable outcome.

## Introduction

Total joint replacement surgery is a very effective treatment option in the end stages of hip and knee osteoarthritis (OA) [1]. By 2009, the numbers of patients undergoing total hip arthroplasty (THA) and total knee arthroplasty (TKA) have risen up to 1.6 and 1.2 per 1,000 per year in Western countries [2]. In the Netherlands, the annual number of THA and TKA is 50,000 [3]. These numbers are expected to further increase in the coming years, due to the aging society and the growing prevalence of obesity [3]. Although the outcomes of THA and TKA are in general favorable with respect to pain, daily activities, and health-related quality of life (HRQoL), 7–34 % of patients are not satisfied with the result of surgery [4].

One of the factors being associated with a poor outcome after THA and TKA concerns the presence of comorbidity [5]. In patients with hip or knee OA in general, comorbidity was found to be highly prevalent and affecting physical functioning and HRQoL [6–8]. Regarding the presence of comorbidity in patients with hip or knee OA undergoing THA or TKA, any form of comorbidity was reported in 73 % of 893 Finnish patients waiting for THA or TKA [9]. Concerning the prevalence of specific comorbidities, the rates in THA and TKA patients vary among recent studies: hypertension in 18–64 % [10–14]; heart failure in 6–32 % [12, 13, 15–17]; diabetes in 4–24 % [9–14, 16, 17]; chronic obstructive pulmonary disease (COPD) in 6–13 % [10, 11, 16, 17]; and back pain in 31–37 % [12, 13, 15] of patients with THA and/or TKA.

With respect to the association of the number of comorbidities and the outcomes of THA and TKA, it was found that an increasing number of affected joints was associated with worse postsurgical pain, physical function, and mental health [15, 18] as well as HRQoL [18, 19]. With respect to the association of specific comorbidities and outcomes of THA and TKA, the presence of neck, ankle/feet/toes pain [17], back pain [20] and stroke [21] in TKA and hypertension [22] in THA, obesity [22], and heart disease [16, 17] in both THA and TKA was associated with a worse health status after surgery. In all of these studies, the outcomes were determined 1–5 years after surgery.

So far, the literature on the associations of comorbidity with outcome in patients with hip or knee OA undergoing THA or TKA often focused on one or a limited number of comorbidities [17, 20, 23]. In other publications, an average comorbidity score was provided [15, 18, 19, 24], which obscures the relationship between specific comorbidities and the patients’ health status. Moreover, regarding the outcomes of THA and TKA, pain and physical functioning rather than HRQoL were considered [15, 19].

In daily practice, orthopedic surgeons take the presence of comorbidity into account in their decision for surgery, in part by using the ASA (American Society of Anesthesiologists) classification to identify patients with an increased risk of death or surgical complications. This use of this classification is, however, not aiming to identify patients with an increased risk of a worse functional outcome [25]. In 2012, 65 % of the patients undergoing THA or TKA in the Netherlands were classified as ASA II (mild systemic disease) and 11 % as ASA III–IV (severe systemic disease with or without constant threat of life) [4]. These figures indicate that comorbidity, both systemic as well as affecting specific organs, is often present in both THA and TKA patients and may influence postoperative outcomes. In addition to the ASA classification, the Charnley Classification [26, 27] is often used; however, this system does not specify specific forms of comorbidity.

Given the lack of knowledge on the incidence and associations of a wide range of specific comorbidities and health status after THA and TKA, the aim of this study was to determine the burden of comorbidities among patients who underwent THA and TKA and to evaluate the association between comorbidities and pain, physical function and HRQoL one year after surgery.

## Method

### Study design

This study had a cross-sectional design. It was a part of multicenter survey concerning the use of physical therapy [28] among consecutive patients who underwent THA or TKA in 2011 in 4 hospitals (Leiden University Medical Center, Leiden; Rijnland Hospital, Leiderdorp; Groene Hart Hospital, Gouda; and Reinier de Graaf Hospital Delft, the Netherlands). The study was carried out from July to October 2012, with a minimum of 7 months and a maximum of 22 months after surgery. This study was set up as a survey to be completed once and therefore judged to fall outside the remit of the Dutch law on Medical Research Involving Human Subjects Act (WMO), and an exemption for medical ethical review was given by the Medical Ethical Committee of the Leiden University Medical Center after checking the precision of the protocol. The study was conducted in accordance with the Handbook for Good Clinical Research Practice of the World Health Organization and Declaration of Helsinki principles [http://www.wma.net/en/30publications/10policies/b3/].

### Recruitment of patients

Patients diagnosed with hip or knee OA were eligible for the study if they were 18 years or older and underwent primary THA or TKA between January 1 and December 31, 2011.

Exclusion criteria were surgery for diagnoses other than OA and revision surgery.

We aimed to obtain total number of 400 completed questionnaires (200 THA and 200 TKA). Anticipating a response rate of 40 %, we planned to invite 1,000 patients from 4 different hospitals. Selection was done by means of hospital registries. All patients in whom the diagnosis primary THA or TKA was made between January 1 and December 31, 2011 were first selected, subsequently, all patients with a diagnosis of hip or knee OA, and age 18 years or older were identified. In hospital 1, all eligible persons were invited. In hospital 2, the survey was sent to all persons who were already participating in a prospective study and underwent surgery in 2011, whereas in hospitals 3 and 4, all consecutive patients who underwent surgery were invited, until the total number of 1,000 invited patients was reached.

In all 4 hospitals, the treating orthopedic surgeon sent a letter explaining the study and requesting the patient to participate, together with an information leaflet, an informed consent form, the survey and a pre-stamped, pre-addressed envelope to all selected patients. After patients signed the informed consent form, they were included in the study. Because the questionnaires were completed anonymously, no information was obtained on the patients who did not respond.

### Sociodemographic and personal characteristics

Sociodemographic characteristics included age (years), sex, level of education (low, medium and high), and marital status (living alone, yes/no). In addition, patient’s height (cm) and weight (kg) were recorded (to calculate the Body Mass Index), as well as their smoking status (current smoker yes/no).

### Presence of comorbidities

Information on comorbidities was gathered with two questionnaires:

First, a comorbidity questionnaire developed by the Dutch Central Bureau of Statistics (CBS) [29] was used, asking for the presence or absence of 19 different comorbidities in the previous year, divided in three domains: *Musculoskeletal comorbidities:* severe back pain (including slipped disc); severe neck or shoulder pain; severe elbow, wrist or hand pain; other chronic rheumatic diseases; *Non*-*musculoskeletal comorbidities:* asthma or COPD (Chronic Obstructive Pulmonary Disease); (severe) cardiac disorder or coronary disease; arteriosclerosis (abdomen or legs); hypertension; (consequences of) stroke; severe bowel disorder; diabetes mellitus; migraine; psoriasis; chronic eczema; cancer; incontinence of urine; *Sensory impairments: *hearing impairments (group and face-to-face conversation); vision impairments (short and long distance); dizziness in combination with falling.

Secondly, we used the self-reported Charnley Classification [26, 27], which consists of three categories: Patients are assigned to class A if they have single joint arthropathy and no significant medical comorbidity. Class B patients have one other joint in need of an arthroplasty, or an unsuccessful or failing arthroplasty in another joint, while class C patients have multiple joints in need of arthroplasty, multiple failing arthroplasties, or significant medical or psychological impairment.

### Physical functioning, pain, and HRQoL

Information on current postoperative physical functioning was collected by means of the pain and physical functioning subscales of the Hip disability Osteoarthritis Outcome Score (HOOS) [30] and the Knee injury Osteoarthritis Outcome Score (KOOS) [31]. The pain subscale consists of 10 and 9 (different) items in the HOOS and KOOS, respectively, whereas the physical functioning subscale comprises the same 17 items regarding activities in daily life in both HOOS and KOOS. The score range is 0–100, with higher scores meaning less pain and better physical functioning.

Health-related quality of life (HRQoL) was measured with the Short Form-36 questionnaire (SF36) [32]. The SF36 contains 8 subscales (physical functioning; role limitations due to physical health problems; bodily pain; general health perceptions; vitality; social functioning; role limitations due to emotional problems; and general mental health). Scores on the subscales were transformed into two component scores: a mental and physical component summary score. By using data from a Dutch general population, norm-based scores (relative to an average score in the general population of 50) were calculated [32]. For all subscale scores and summary scores, the score range was 0–100, with higher scores indicating a better health status.

### Statistical analyses

Patients’ characteristics, the presence of different specific comorbidities, pain, physical functioning, and HRQoL were analyzed by using descriptive statistics. Differences between the characteristics of patients undergoing THA and TKA were examined by means of unpaired Students’ *t* test or Chi-square tests, where appropriate.

To study the relationship between the number of comorbidities and the four outcomes, multivariate regression models were employed. For this purpose, the number of comorbidities was categorized into four groups: 1–2 comorbidities, 3–4 comorbidities, and 5 or more comorbidities, with 0 comorbidities being the reference group.

Then, all comorbidities (which occured in ≥5 of the patients) and potential confounders which were significantly associated with the outcomes were included in final multivariate regression models.

All the analyses were corrected for BMI, whereas the analyses with pain and physical functioning as dependent variables were also adjusted for sex and age (SF36 scores were calculated using norm data so the correction for age and sex would be redundant).

All data were analyzed using the SPSS statistical package (version 20.0, SPSS, Chicago, Illinois). The level of statistical significance was set at *p* < 0.05 for all analyses.

## Results

### Study population

In total 1,005 patients were requested to complete the survey in the 4 participating hospitals. Of these patients, 539 (53.6 %) underwent THA and 466 (46.4 %) TKA. The response to the survey was 521 out of 1,005 (51.8 %) for the total group, with 281 out of 539 (52.1 %) patients responding in the THA and 240 out of 466 (51.5 %) in the TKA groups.

Table 1 shows the baseline characteristics of the study population. Two-thirds of the patients were female (65.2 %), their mean age was 70.0 (SD 9.3) years, and the mean BMI 27.8 (SD 4.7), with the latter being significantly lower in the THA group as compared to the TKA group (26.7 (SD 4.1) and 29.2 (SD 5.1), respectively (*p* < 0.001)). There were significantly more obese (BMI 30–40) patients in the TKA group than in the THA group (36.6 % and 17.9 %, respectively, *p* < 0.001), while no difference regarding the frequency of morbid obesity (BMI > 40) was seen between THA and TKA.

**Table 1:** Characteristics of 521 patients who underwent total hip arthroplasty or total knee arthroplasty in 2011

Variable	All patients (*N* = 521)	Total hip (*N* = 281)	Total knee (*N* = 240)	*p* value
Sex, female (*N* = 515)	336 (65.2 %)	178 (64.0 %)	158 (66.7 %)	0.53
Age, years (mean, SD)	70.0 (9.3)	69.8 (9.5)	70.1 (9.0)	0.69
Body Mass Index (BMI) (mean, SD) (*N* = 508)	27.8 (4.7)	26.7 (4.1)	29.2 (5.1)	<0.001*
BMI < 25	150 (29.5 %)	100 (36.6 %)	50 (21.3 %)	<0.001*
BMI 25–30 (overweight)	217 (42.7 %)	123 (45.1 %	94 (40.0 %)	0.25
BMI 30–40 (obesity)	135 (26.6 %)	49 (17.9 %)	86 (36.6 %)	<0.001*
BMI > 40 (morbid obesity)	6 (1.2 %)	1 (0.4 %)	5 (2.1 %)	0.07
Current smoker (*N* = 507)	44 (8.7 %)	29 (10.5 %)	15 (6.5 %)	0.11
Education level (*N* = 400)				
Low	160 (40.0 %)	82 (38.5 %)	78 (41.7 %)	0.51
Medium	151 (37.8 %)	76 (35.7 %)	75 (40.1 %)	0.36
High	89 (22.2 %)	55 (25.8 %)	34 (18.2 %)	0.07
Marital status, living alone (*N* = 365)	109 (29.9 %)	55 (28.8 %)	54 (31.0 %)	0.64
HOOS or KOOS pain; mean (SD)	81.7 (19.1)	84.0 (17.1)	79.0 (20.9)	0.010*
HOOS or KOOS physical functioning; mean (SD)	78.9 (20.9)	81.3 (18.9)	76.1 (22.7)	0.014*
SF36 physical component summary scale; mean (SD)	45.4 (8.6)	45.6 (8.9)	45.2 (8.2)	0.61
SF36 mental component summary scale; mean (SD)	47.7 (7.7)	48.0 (7.6)	47.5 (7.8)	0.53
Charnley Classification (*N* = 505)				
Class A	125 (24.8 %)	81(29.9 %)	44 (18.8 %)	0.004*
Class B	102 (19.6 %)	56 (19.9 %)	46 (19.2 %)	0.78
Class C	278 (55.0 %)	134 (49.4 %)	144 (61.5 %)	0.006*

All variables expressed as numbers (%), unless stated otherwise

* Statistical significant difference between total hip and total knee patients by means of a Student *t* test or Chi-square test where appropriate

The average pain and physical functioning scores as measured by the HOOS and KOOS, respectively, were significantly higher in the THA group than in the TKA group (*p* = 0.010 and *p* = 0.014, respectively), whereas the SF36 mental and physical component summary scales did not differ between THA and TKA.

In the group of patients who underwent THA, statistically significantly more patients were classified as Charnley Class A and significantly less patients classified as Charnley Class C as compared to TKA (*p* = 0.004 and *p* = 0.006, respectively).

Concerning the occurrence of specific comorbidities, hypertension and hearing impairments in a group conversation were the most frequently reported comorbidities (>25 % for the total group). Severe back pain; severe neck/shoulder pain; severe elbow, wrist or hand pain; cancer; incontinence of urine; and vision impairment short distances were reported by 15–25 % of the patients in the total group (Table 2).

**Table 2:** Comorbidities in 521 patients who underwent total hip arthroplasty or total knee arthroplasty in 2011

Variable	All patients (*N* = 521)	Total hip (*N* = 281)	Total knee (*N* = 240)	*p* value
Musculoskeletal comorbidities				
Severe back pain (hernia included)	97 (18.6 %)	46 (16.4 %)	51 (21.3 %)	0.15
Severe neck/shoulder pain	101 (19.4 %)	50 (17.8 %)	51 (21.2 %)	0.32
Severe elbow, wrist or hand pain	86 (16.5 %)	43 (15.3 %)	43 (17.9 %)	0.42
Other rheumatic diseases	65 (12.5 %)	30 (10.7 %)	35 (14.6 %)	0.18
Non-musculoskeletal comorbidities				
Asthma or COPD	59 (11.3 %)	29 (10.3 %)	30 (12.5 %)	0.43
(Severe) cardiac disorder or coronary disease	55 (10.6 %)	31 (11.0 %)	24 (10.0 %)	0.70
Arteriosclerosis in abdomen and legs	32 (6.1 %)	18 (6.4 %)	14 (5.8 %)	0.79
Hypertension	220 (42.2 %)	108 (38.4 %)	112 (46.7 %)	0.06
(Consequences) of a stroke	31 (6.0 %)	14 (5.0 %)	17 (7.1 %)	0.31
Severe bowel disorder	23 (4.4 %)	10 (3.6 %)	13 (5.4 %)	0.30
Diabetes Mellitus	70 (13.4 %)	31 (11.0 %)	39 (16.2 %)	0.08
Migraine	33 (6.3 %)	16 (5.7 %)	17 (7.1 %)	0.52
Psoriasis	16 (3.1 %)	9 (3.2 %)	7 (2.9 %)	0.85
Severe eczema	21 (4.0 %)	12 (4.3 %)	9 (3.8 %)	0.76
Cancer	90 (17.3 %)	38 (13.5 %)	52 (21.7 %)	0.01*
Incontinence of urine	96 (18.4 %)	53 (18.9 %)	43 (17.9 %)	0.78
Sensory impairments				
Hearing impairments (group conversation)	139 (26.7 %)	68 (24.2 %)	71 (29.6 %)	0.17
Hearing impairments (face-to-face conversation)	20 (3.8 %)	9 (3.2 %)	11 (4.6 %)	0.41
Vision impairments (short distances)	94 (18.0 %)	52 (18.5 %)	42 (17.5 %)	0.77
Vision impairments (long distances)	45 (8.6 %)	30 (10.7 %)	15 (6.2 %)	0.07
Dizziness in combination with falling	32 (6.1 %)	15 (5.3 %)	17 (7.1 %)	0.41
Number of comorbidities				
0	72 (13.8 %)	45 (16.0 %)	27 (11.2 %)	0.12
1 or 2	232 (44.5 %)	130 (46.3 %)	102 (42.5 %)	0.39
3 or 4	133 (25.6 %)	69 (24.5 %)	64 (26.7 %)	0.58
≥5	84 (16.1 %)	37 (13.2 %)	47 (19.6 %)	0.047*

* Statistical significant difference between total hip and total knee patients by means of a Chi-square test

The occurrence of cancer was significantly lower in the THA group than in the TKA group [38 (13.5 %) and 52 (21.7 %), respectively (*p* = 0.01)]. Regarding all other specific comorbidities, there were no differences between THA and TKA (Table 2).

Table 2 also shows the distribution of the numbers of comorbidities in the total group and the THA and TKA groups. Overall, 86.2 % of the patients had one or more comorbidities. The proportion of patients with 5 or more comorbidities was higher in patients with TKA as compared to THA [47 (19.6 %) and 37 (13.2 %), respectively (*p* = 0.047)].

### Associations of the number of specific comorbidities with pain, physical functioning, and HRQoL

Table 3 shows that in THA, and adjusted for age, sex and BMI, where appropriate, the presence of 1 or 2 comorbidities was significantly associated with worse physical functioning relative to no comorbidities. The presence of 3 or 4, and ≥5 comorbidities was associated with more pain, worse physical functioning, and a worse score on the physical component summary scale of the SF 36.

**Table 3:** Associations between the number of comorbidities and pain, physical functioning, and quality of life in 521 patients after THA and TKA

	Pain (H/KOOS subscale)^a^		Physical functioning (H/KOOS subscale)^a^		Quality of life (SF36 physical component summary scale)^b^		Quality of life (SF36 mental component summary scale)^b^	
	Mean (SD)	B (95 % CI)	Mean (SD)	B (95 % CI)	Mean (SD)	B (95 % CI)	Mean (SD)	B (95 % CI)
Number of comorbidities in 281 patients after THA								
0	93.6 (8.7)		93.5 (9.3)		49.7 (9.7)		49.8 (4.4)	
1 and 2	86.5 (17.0)	−6.6 (−13.7; 0.4)	84.3 (18.1)	−8.6 (−16.4; −0.9)*	46.6 (8.4)	−2.9 (−6.2; 0.4)	48.2 (7.8)	−1.7 (−4.7; 1.3)
3 and 4	79.8 (17.0)	−12.8 (−20.9; −4.7)*	77.3 (18.0)	−14.9 (−23.8; −6.1)*	42.9 (8.1)	−6.5 (−10.2; −2.8)*	47.0 (8.2)	−3.0 (−6.3; 0.4)
5 and more	76.1 (18.1)	−15.9 (−25.3; −6.4)*	69.2 (21.7)	−22.1 (−32.4; −11.7)*	41.8 (7.9)	−7.1 (−11.7; −2.5)*	46.5 (9.2)	−3.8 (−7.9; 0.3)
Number of comorbidities in 240 patients after TKA								
0	76.2 (20.7)		66.5 (23.0)		48.7 (6.4)		48.7 (8.8)	
1 and 2	84.3 (18.7)	7.3 (−3.3; 17.8)	83.0 (18.4)	16.1 (4.8; 27.5)	47.3 (7.5)	−1.2 (−5.5; 3.1)	49.5 (5.8)	1.1 (−2.8; 5.1)
3 and 4	74.9 (22.8)	−1.2 (−12.7; 10.2)	75.5 (23.6)	10.0 (−2.4; 22.3)	43.9 (8.6)	−4.5 (−9.2; 3.1)	46.5 (9.3)	−1.7 (−6.0; 2.7)
5 and more	74.6 (21.0)	−1.6 (−13.4; 10.1)	65.7 (24.8)	−0.2 (−13.0; 12.5)	41.7 (8.6)	−6.7 (−11.4; −2.0)*	44.6 (7.6)	−3.6 (−8.0; 0.8)

* Statistical significance (*p* < 0.05)

^a^Statistical analyses by means of a multivariate regression analyses adjusted for age, sex, and BMI

^b^Statistical analyses by means of a multivariate regression analyses adjusted for BMI

Table 3 shows that in TKA, only the presence of ≥5 comorbidities was associated with a worse score regarding the physical component summary scale of the SF 36.

The presence of all the 19 specific comorbidities, except hypertension, was associated with one or more of the outcomes in both THA and TKA (results not shown).

### Multivariate regression models with all comorbidities and pain, physical functioning, and HRQoL

Table 4 shows the results of the final multivariate regression models for worse outcome in THA for all four outcomes. Comorbidities included in 3 out of 4 of the association models were dizziness in combination with falling and severe back pain (associated with more pain and worse physical functioning and physical component of HRQoL). Comorbidities included in 2 out of 4 of the models were severe back pain and arteriosclerosis in abdomen or legs (associated with more pain and worse physical functioning).

**Table 4:** Comorbidities included in the final multivariate regression models of the association of comorbidity with pain, physical functioning, and health-related quality of life in 281 patients undergoing THA

	B (95 % CI)	*p* value	*R* ^2^
HOOS/KOOS subscale pain^a^			
Arteriosclerosis in abdomen or legs	−12.3 (−21.7; −2.9)	0.010	0.147
Dizziness in combination with falling	−12.1 (−23.5; −0.7)	0.037	
Cardiac disorders	−10.6 (−17.6; −3.6)	0.003	
Severe back pain	−8.0 (−14.0; −2.0)	0.009	
Cancer	−6.7 (−12.6; −0.7)	0.028	
HOOS/KOOS subscale physical functioning^a^			
Dizziness in combination with falling	−22.0 (−34.2; −9.9)	<0.001	0.215
Arteriosclerosis in abdomen or legs	−14.9 (−25.3; −4.5)	0.005	
Severe back pain	−12.9 (−19.3; −6.4)	<0.001	
Asthma or COPD	−9.0 (−16.8; −1.3)	0.023	
Cancer	−8.0 (−14.3; −1.7)	0.013	
SF36 physical component scale^b^			
Dizziness in combination with falling	−6.4 (12.5; −0.4)	0.037	0.094
Severe back pain	−3.5 (−6.6; −0.4)	0.028	
Incontinence of urine	−3.2 (−6.4; −0.1)	0.045	
Hypertension	−2.9 (−5.3; −0.5)	0.017	
SF36 mental component scale^b^			
(Consequences of) a stroke	−4.8 (−9.0; −0.6)	0.024	0.044
Severe elbow, wrist or hand pain	−3.2 (−6.2; −0.3)	0.032	

^a^Statistical analyses adjusted for age, sex, and BMI

^b^Statistical analyses adjusted for BMI

In Table 5, the results for TKA are shown. Vision impairments (long distances) were included in 3 out of 4 of the association models and associated with more pain and worse physical functioning and mental component of HRQoL. Comorbidities included in two out of four association models were dizziness in combination with falling (associated with more pain and worse physical functioning) and severe elbow, wrist or hand pain (associated with worse physical functioning and physical component of HRQoL).

**Table 5:** Comorbidities included in the final multivariate regression models of the association of comorbidity with pain, physical functioning, and health-related quality of life in 240 patients undergoing TKA

	B (95 % CI)	*p* value	*R* ^2^
HOOS/KOOS subscale pain^a^			
Dizziness in combination with falling	−17.2 (−29.2; −5.2)	0.005	0.068
Vision impairments (long distances)	−12.0 (−24.0; −0.1)	0.049	
HOOS/KOOS subscale physical functioning^a^			
Dizziness in combination with falling	−24.4 (−37.4; −11.4)	<0.001	0.174
Vision impairments (long distances)	−16.9 (−29.2; −4.6)	0.007	
Severe elbow, wrist or hand pain	−9.1 (−17.1; −1.0)	0.028	
Asthma or COPD	12.5 (3.2; 21.9)	0.009	
SF36 physical component scale^b^			
Incontinence of urine	−4.9 (−8.3; −1.6)	0.004	0.107
Severe elbow, wrist or hand pain	−4.2 (−7.3; −1.1)	0.007	
SF36 mental component scale^b^			
Diabetes	−3.3 (−6.1; −0.5)	0.006	0.090
Migraine	−4.1 (−8.2; −0.1)	0.046	
Vision impairments (short distances)	−4.3 (−7.3; −1.2)	0.006	

^a^Statistical analyses adjusted for age, sex, and BMI

^b^Statistical analyses adjusted for BMI

## Discussion

This study in patients who underwent THA or TKA showed the presence of considerable amount of comorbidities in patients after surgery. Hypertension and hearing impairments in a group conversation had the highest occurrence rates (>25 %), while severe back pain; neck/shoulder pain; elbow, wrist or hand pain; cancer; and vision impairments regarding short distances were also relatively frequent, as they were reported by 15–20 % of the patients.

Some of the present frequencies of the occurrence of specific comorbidities in this study were comparable with earlier studies, i.e., hypertension (42 % in THA [13] and 42 % in TKA [12]); heart disease (7 % in THA [16] and 8 % in TKA [10]); diabetes (10 % in THA [13] and 11 % in TKA [12]); and COPD (9 % in THA [16] and 10 % in TKA [10, 12]).

The rate of the presence of back pain in the present study was somewhat lower than in earlier studies (37 % in THA [13] and 31–35 % in TKA [12, 17]), probably due to the fact that we asked for *severe* back pain only. In general, comparisons of rates with previous studies are hampered by the differences in time lapse between surgery and the measurement of comorbidities and outcomes.

In our study, an increasing number of comorbidities was associated with worse pain and physical function, and reduced quality of life. This observation appeared to be somewhat stronger in THA than in TKA. This finding was also seen for HRQOL and/or or physical functioning in studies by Rat et al. [19] and Stevens et al. [18], concerning THA and TKA plus THA, respectively. Perrucio et al. [15] reported more pain and worse physical functioning with increasing joint counts in patients after TKA. This is in some extent comparable with our study, in which multiple sites of joint involvement were included as specific comorbidities. However, no good comparisons can be made since no specified analyses for other impaired joints were conducted in our study.

The present study focused on 19 different comorbidities. From models including all comorbidities, it appeared that in THA, dizziness with falling and severe back pain were associated with 3 out of 4 outcomes, while in TKA, dizziness with falling and vision impairments (long distances) were associated with 2 out of 4 outcomes.

The presence of dizziness in combination with falling is difficult to compare with other studies, as these studies did not include this comorbidity. Dizziness has, however, been previously identified as one of the main reasons for a longer stay in hospital after surgery [33] and can be related to presence of anemia, which is related to worse outcome in hip fracture patients [15]. In our study, severe back pain was also consistently associated with worse pain and physical function, and reduced quality of life in THA. This finding is in contrast with the results from the study by Novicoff et al. [20] in which this association was found in TKA.

In our study, obesity was not considered as a specific comorbidity, but rather as an influential factor. Therefore, BMI was included in all analyses. The literature shows conflicting evidence regarding the association of obesity with complications after surgery [34]. Regarding physical functioning, however, there seems to be no difference in outcome between obese and non-obese patients. Only morbidly obese patients (BMI > 40) were found to be at a greater risk for perioperative complications such as infection and revision than patients with a BMI < 40, most likely due to the additional other existing comorbidities (i.e., diabetes, hypertension, and cardiac diseases) [35]. On the other hand, obesity can be considered as a contraindication by orthopedic surgeons in their decision for surgery, based on the ASA criteria, thereby leading to a probable underestimation of their influence on postsurgical outcome. It remains unclear to what extent this occurred in our study, as the frequency of morbid obesity (BMI > 40) was only 1 % in our study population.

This study has a number of limitations. First, only 4 hospitals in a specific area in the Netherlands were involved, the sample size was limited and varied between sites, the response rate was moderate (52 %), and no comparison of the characteristics of the participants and non-responders could be made. Therefore, results cannot be generalized to all patients undergoing THA or TKA. However, based on baseline characteristics, it seems to be a representative sample of this patient group. Furthermore, the study is cross-sectional and not longitudinal, which makes that only associations can be determined, and no predictions can be made. Preferably, data on the presence of comorbidity should be gathered preoperatively. Moreover, the assessments only included self-reported questionnaires. Examination of the medical records could probably have given more reliable and additional information; however, this very time-consuming method could not be used in the framework of this study.

Despite these limitations, this study taking a large number of comorbidities into consideration and analyzing THA and TKA patients separately found that not only the number of comorbidities but also their nature are related to the outcomes. In particular, dizziness in combination with falling plays a consistent role in both THA and TKA, and severe low back pain in THA. Although a prospective study including the presence of a wide range of comorbidities preoperatively is needed, the results of the present study suggest that a large number of specific comorbidities have an impact on patients’ health status after surgery. As many comorbidities are chronic by nature and therefore likely to have been present before surgery as well, their presence should probably be ascertained before surgery and treated if possible, since the predictive value is confirmed in earlier studies [36]. Although not yet confirmed in a prospective study, in particular, the presence of dizziness in combination with falling should probably be taken into account before surgery.

Currently, the ASA classification [25] is used preoperatively, but this is mainly aimed at the selection of patients regarding their risk to undergo surgery, and not with respect to their risk of adverse functional outcomes. With respect to the latter, the Charnley Classification is a commonly used instrument [26, 27], but it does not distinguish clearly between specific conditions. Detecting the presence of more specific comorbidities seems to be of additional value.
